# Evaluating the Effects of Cutoffs and Treatment of Long-range Electrostatics in Protein Folding Simulations

**DOI:** 10.1371/journal.pone.0039918

**Published:** 2012-06-29

**Authors:** Stefano Piana, Kresten Lindorff-Larsen, Robert M. Dirks, John K. Salmon, Ron O. Dror, David E. Shaw

**Affiliations:** 1 D. E. Shaw Research, New York, New York, United States of America; 2 Center for Computational Biology and Bioinformatics, Columbia University, New York, New York, United States of America; Bioinformatics Institute, Singapore

## Abstract

The use of molecular dynamics simulations to provide atomic-level descriptions of biological processes tends to be computationally demanding, and a number of approximations are thus commonly employed to improve computational efficiency. In the past, the effect of these approximations on macromolecular structure and stability has been evaluated mostly through quantitative studies of small-molecule systems or qualitative observations of short-timescale simulations of biological macromolecules. Here we present a quantitative evaluation of two commonly employed approximations, using a test system that has been the subject of a number of previous protein folding studies–the villin headpiece. In particular, we examined the effect of (i) the use of a cutoff-based force-shifting technique rather than an Ewald summation for the treatment of electrostatic interactions, and (ii) the length of the cutoff used to determine how many pairwise interactions are included in the calculation of both electrostatic and van der Waals forces. Our results show that the free energy of folding is relatively insensitive to the choice of cutoff beyond 9 Å, and to whether an Ewald method is used to account for long-range electrostatic interactions. In contrast, we find that the structural properties of the unfolded state depend more strongly on the two approximations examined here.

## Introduction

Recent years have seen dramatic increases in the attainable lengths of molecular dynamics (MD) simulations, which have been made possible by improvements in both algorithms and computer hardware [1]–[5]. The computational cost associated with such simulations is still very large, however, representing a significant obstacle to the more widespread application of MD simulation techniques to the study of complex biological processes [6], [7].

MD simulations, like many other computational methods, face a tradeoff between computational efficiency and accuracy. In order to perform MD simulations less expensively or on longer timescales, a number of approximations of the potential energy function are often employed. Systematic studies to assess the effect of introducing such approximations have generally been limited by the availability of computational resources, and have for the most part focused on either the quantitative characterization of small-molecule systems or more qualitative investigations of larger biomolecules.

The most computationally expensive part of an MD simulation is generally the calculation of nonbonded forces, including both electrostatic and van der Waals interactions, which act between all pairs of atoms. A common approach to reduce the cost of this computation is to ignore any interaction between atoms separated by more than some cutoff distance. This approach is generally accepted as being sufficiently accurate for van der Waals forces, which decay rapidly to zero as the distance increases. Electrostatic forces, however, fall off much more slowly with distance, and a simple truncation at the cutoff distance may introduce substantial errors. A number of approximations have been proposed that modify the electrostatic potential so that the forces approach zero or are exactly zero at the cutoff distance [8], thus partially alleviating the severity of the artifacts introduced by the truncation. An alternative approach is to fully account for the long-range component of the electrostatic interactions, which is most often achieved using various Ewald summation techniques [9]. This technique involves splitting the electrostatic interactions into a quickly decaying near component that can be calculated for all atom pairs within a fixed cutoff–typically the same cutoff length used for van der Waals forces–and a long-range component that can be more efficiently calculated using other methods (e.g., using Fourier transforms in conjunction with periodic boundary conditions). While such Ewald methods involve a cutoff distance, the choice of cutoff acts to shift the computational burden between the near and long-range calculations, without limiting the accuracy of the calculated forces. This differs from pure cutoff-based schemes for electrostatics and van der Waals forces, which ignore interactions beyond the cutoff and thus decrease in accuracy as the cutoff becomes smaller.

There is evidence in the literature that both the method used for treating long-range electrostatics and the choice of cutoff distance (for cutoff-based electrostatic and van der Waals interactions) may affect the utility of MD simulations for studying biological systems, with some schemes being more accurate than others [8]–[16]. In the area of protein folding simulations, for example, we recently observed that a double-norleucine mutant of villin [17] has an effective melting temperature of ∼380 K in simulations employing the Amber ff03/TIP3P force field [18], an Ewald method to account for the long-range electrostatics, and a 9.0-Å cutoff for van der Waals forces [19]. On the other hand, Pande and coworkers reported a melting temperature of ∼300 K in simulations using the same force field, but employing a reaction field method–which is cutoff-based–for electrostatics, with a cutoff of 8.0 Å [20], [21]. Although there were other differences in the simulations and analysis, it appears plausible that at least some of the large disparity in the calculated stability may have arisen from differences in cutoff length or the treatment of long-range electrostatic effects [22].

Prompted by such observations of how the treatment of nonbonded interactions may affect simulation results, here we use long MD simulations performed on Anton, a special-purpose computer for MD simulations [23], to examine and quantify how different schemes for the approximation of nonbonded interactions affect the results of protein folding simulations. Overall, we find that the free energy of the folding of a small protein is rather insensitive even to relatively radical approximations, whereas the structural properties of the unfolded state depend more strongly on the scheme and parameters employed.

## Methods

We chose folding simulations of a fast-folding variant of the villin headpiece domain [17] with the CHARMM22* force field [19], [24] as our test system for MD simulations. This system includes the villin headpiece (a protein domain with 35 amino acids), 4,397 water molecules and 5 ions, for a total of 13,773 atoms in a 52-Å cubic box. It is sufficiently complex to capture many of the important aspects of biological systems, yet it is sufficiently small and its kinetics are sufficiently fast to allow for efficient simulation of folding on Anton, thus making it possible to obtain statistically meaningful estimates of structural and thermodynamic quantities. We performed 14 simulations of the folding and unfolding of villin, distributed as follows: (i) seven simulations were performed with atom-based cutoffs ranging between 8.0 and 12.0 Å and the *k*-space Gaussian split Ewald (GSE) method [25] for the treatment of long-range electrostatics and (ii) seven simulations were performed with the same set of atom-based cutoffs, but using a cutoff-based force-shifting technique (SHIFT) [8], in which a constant is added to the electrostatic force between atoms so that the net force is zero at the cutoff distance and electrostatic interactions beyond the cutoff distance are ignored. In all simulations we used the same cutoff for both the electrostatics and the van der Waals interactions, with the van der Waals interactions modeled by Lennard-Jones terms truncated at the cutoff. Except where otherwise noted, simulations used a 32×32×32 mesh for long-range electrostatics. The remaining parameters of the GSE method were adjusted in each simulation to minimize the root mean square (rms) error in computed forces; the rms errors due to the GSE scheme ranged between 1.2×10^−2^ kcal·mol·Å^−1^ for the 8-Å cutoff simulation and 7×10^−4^ kcal·mol·Å^−1^ for the 12-Å cutoff simulation (see Results and Discussion for a further discussion of the effect of this error on the results). Since the accuracy of electrostatic forces in the GSE calculations are largely independent of the choice of cutoff, the simulations using GSE primarily address the effects of changing the van der Waals cutoff distance, whereas the simulations using SHIFT reflect the changing accuracy of both van der Waals and electrostatic terms. For the purpose of comparison, the GSE simulation with a cutoff of 12 Å is treated as the most accurate computational result.

MD simulations in the NVT ensemble were performed and analyzed as recently described [19], [26]. The systems were coupled to a Nosé-Hoover thermostat [27], [28] with a reference temperature of 360 K and a relaxation time of 10 ps. The equations of motion were integrated using a RESPA scheme [29] with time steps of 5.0 fs for the long-range electrostatic interactions and 2.5 fs for all other interactions. Bonds involving hydrogen atoms were constrained to their equilibrium length using the M-SHAKE algorithm [30]. Each simulation was between 21 and 125 µs long, for a total of 578 µs of simulation. We also performed control simulations in the NPT ensemble [31] with GSE and cutoffs of 9.0, 9.5, and 10.0 Å, ranging between 25 and 86 µs in length, for a total of 154 µs of simulation. These simulations gave results statistically indistinguishable from the corresponding NVT simulations, and only the NVT results are discussed here. The SHIFT approach was chosen as it has been shown to produce remarkably small errors in the forces compared to an Ewald-based method for the treatment of the long-range interactions [22], yet it is computationally less costly, resulting in a 1.5- to 2-fold performance gain on the 64-node Anton machines used in this study.

The simulation trajectories were partitioned into folded and unfolded segments using a transition-based approach [32], [33] applied to the time series representing rms deviation of C_α_ positions from the crystal structure, as described in refs. 19 and 26. This approach has been shown to produce robust estimates of folding free energies and rates [19], [26]. A total of 339 folding or unfolding events were observed for the 14 simulations, with a minimum of 6 and a maximum of 72 events observed in each simulation.

## Results and Discussion

**Figure 1:**
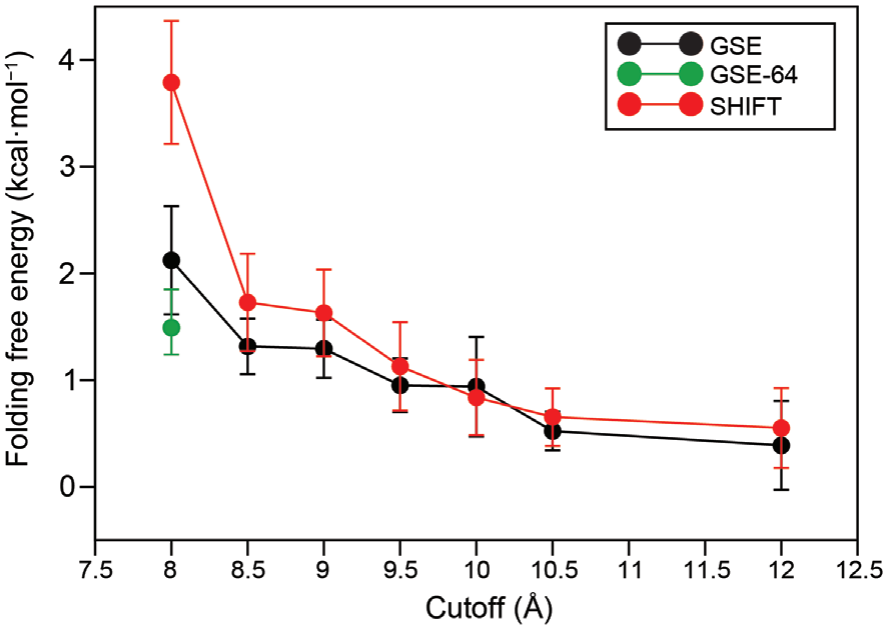
Free energy of folding as a function of cutoff length. The free energies of folding were calculated from the ratio of the populations of the folded and unfolded states and are reported for SHIFT simulations (red), GSE simulations with a 32×32×32 mesh (black) and a 8-Å cutoff GSE simulation with a 64×64×64 mesh (green). Error bars represent the standard error of the mean, estimated using a blocking procedure [38].

By performing the simulations at the experimental melting temperature (360 K, which is close to the melting temperature in simulation for the force field employed) we were able to observe the protein spontaneously fold and unfold multiple times in each simulation. We calculated the fraction of time spent in the folded state, and thereby the free energy of folding, in each of the 14 simulations (Fig. 1).

While the overall structure of the folded state is the same in all cases, we observed the folded state becoming less stable as the cutoff decreased (in both the GSE and SHIFT simulations). At the longest cutoff tested (12.0 Å) we found the folding free energy to be ∼0.5 kcal·mol^−1^ for both GSE and SHIFT simulations, while at the shortest cutoff tested (8.0 Å) the folding free energy was 2 kcal·mol^−1^ for the GSE simulation and 4 kcal·mol^−1^ for the SHIFT simulation. In general, with very short cutoffs, the protein is less stable when using SHIFT than when using GSE, but the two approaches give similar folding free energies for cutoffs above ∼9.0 Å. Part of the large error observed in the short-cutoff GSE simulations could be ascribed to the use of a relatively coarse 32×32×32 mesh. An additional control simulation performed with an 8-Å cutoff and a finer 64×64×64 mesh gives a folding free energy of 1.4±0.1 kcal·mol^−1^; the remaining 1 kcal·mol^−1^ difference with respect to the 12-Å cutoff simulations is probably the result of van der Waals force truncation [34]. All other GSE simulations used a 32×32×32 mesh, as comparisons to forces computed with a finer mesh showed this mesh size to introduce rms force errors less than 10^−2^ kcal·mol^−1^·Å^−1^ for cutoffs above 8 Å. In summary, from the perspective of estimating the folding free energy, we find that (i) for cutoffs ≥9.0 Å there is a modest increase in stability as the cutoff is increased, and (ii) for cutoffs ≥9.5 Å there is very little, if any, difference between using GSE or a force-shifting approach.

**Figure 2:**
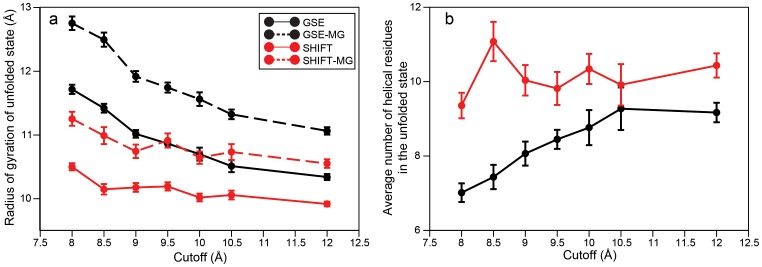
Structural properties of the unfolded state as a function of cutoff length. Panel (a) shows the average radius of gyration in the unfolded state (solid lines) and in molten globule (MG) conformations (dashed lines), where MG conformations are defined as unfolded conformations not containing any secondary structure elements. Panel (b) shows the average number of helical residues in the unfolded state, calculated using STRIDE [39]. Error bars represent the standard error of the mean estimated using blocking.

We now turn our attention to the structural properties of the unfolded state, as these have been shown to be strongly affected by force-field details [19], and we thus expect that they could be more sensitive to the simulation parameters. Indeed, we find that both the radius of gyration and the amount of residual helicity in the unfolded state are influenced by the cutoff and the method used to treat the electrostatics (Fig. 2). In simulations with GSE, the unfolded state becomes more compact as the cutoff is increased. In simulations using SHIFT, the unfolded state is substantially more compact than in simulations using GSE, but the value of the radius of gyration does not depend as strongly on the cutoff as in the GSE simulations. There appears to be a correlation between compactness and the number of helical residues in the unfolded state, with more compact unfolded states also displaying a larger fraction of helical residues. This correlation is not surprising, as α-helix formation is one of the most effective ways to produce compact structures [35]. In order to examine whether the increased helicity is a cause or an effect of the increased compactness, we determined the radius of gyration of “molten globule” conformations (i.e., unfolded state conformations not containing any secondary structure). We found that these conformations, too, typically had a lower radius of gyration in SHIFT simulations than in GSE simulations (Fig. 2a), suggesting that the SHIFT approximation generally increases the hydrophobicity of the protein chain and its tendency to form compact structures. The increased helicity of SHIFT simulations is likely a consequence of this increased compactness, rather than its cause.

**Figure 3:**
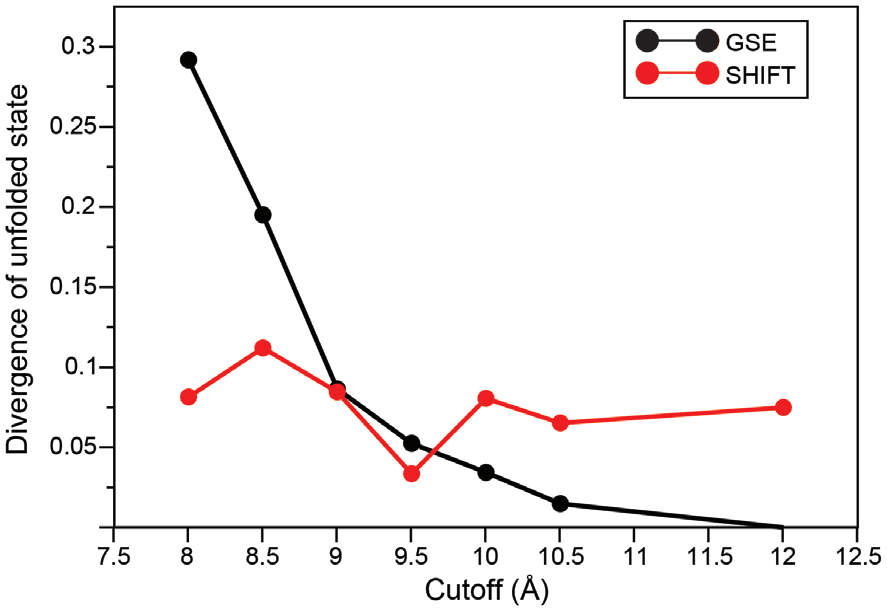
Structural difference of the unfolded states observed in simulations. The plot reports the Kullback-Leibler divergence between the probability densities of the unfolded states projected in a four-dimensional space and the reference probability density (which is based on the GSE simulation performed with a 12-Å cutoff).

To further quantify the structural difference between unfolded states sampled with different electrostatics methods, we projected the unfolded state of each simulation into a four-dimensional space (defined by the radius of gyration and by the number of helical turns formed in each of the three helices) and calculated the Kullback-Leibler divergence from the reference probability distribution (i.e., the GSE simulation performed with a 12-Å cutoff). As a comparison, the calculated divergence between any two of three independent parts of the longest simulation (GSE with 9.5-Å cutoff, total length 126 µs) is ∼0.02. As most simulation lengths are about one-third that of the longest simulation, this number can be taken as an estimate of the limit of statistical accuracy of the divergence estimate. It turns out that the divergence is largest for the short-cutoff simulations and in GSE it progressively decreases when the cutoff is increased (Fig. 3). Interestingly, for very short cutoffs the divergence from the reference simulation is smaller in SHIFT than in GSE calculations, reflecting the fact that SHIFT calculations have a more compact unfolded state, similar to GSE calculations performed with longer cutoffs. In all cases, the differences between the distributions, while statistically significant, are relatively small. This suggests that the simulations are not sampling vastly different regions of conformational space and also indicates that the structural properties of the unfolded state are reasonably well converged in each simulation.

**Figure 4:**
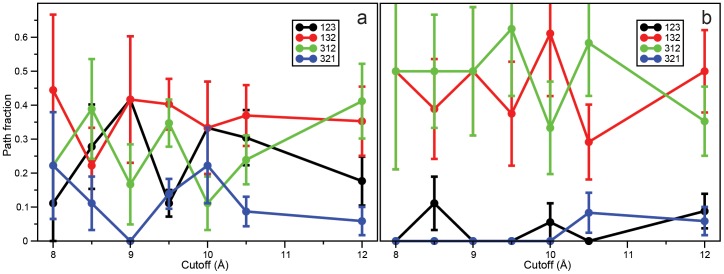
Order of helix formation during folding for GSE (a) and SHIFT (b) simulations with various cutoffs. The four observed orders of formation of the three helices are reported in different colors (see Figure key, where “132” means that helix 1 forms first, helix 3 forms second and helix 2 forms third). Error bars were estimated assuming all folding events were independent.

Finally, it has been shown that the properties of the unfolded state can influence the folding pathway [19], [26], as structural fragments that are more native-like in the unfolded state tend to form first along the folding pathway [26]. For each simulation, we have quantified the order of helix formation during folding, as described in Piana et al. [19]. This metric has been shown to be useful in highlighting differences in folding mechanisms across different force fields. We find that the choice of cutoff has little influence on the relative order of helix formation during folding (Fig. 4). On the other hand, the choice of GSE versus SHIFT does seem to make some difference. In both SHIFT and GSE simulations, either helix 1 or helix 3 can form first with roughly equal probability, in agreement with previous findings [19], but while in GSE simulations helix 2 forms second in a sizable fraction of folding events (Fig. 4a), it almost always folds last in SHIFT simulations (Fig. 4b). This result suggests that while the SHIFT approximation preserves the high-level picture of which piece of the structure forms first during folding, it can still have subtle effects on the details of the folding pathways.

## Conclusions

The use of van der Waals cutoffs or methods that neglect or approximate the long-range contributions to the electrostatic interactions are approaches that are often used to decrease the computational cost of MD simulations. The results presented here provide a quantitative test of these approximations based on long simulations of the folding thermodynamics and structural properties of a small protein. Our results show that different molecular properties are affected differently by the various approximations. The extent to which these approximations can be tolerated thus depends in part on the questions the simulations are intended to answer. We find that short cutoffs or the use of the force-shifting truncating (SHIFT) approximation have the effect of subtly shifting the balance between hydrophobic and hydrophilic interactions such that more compact structures are stabilized. We expect this effect to be rather general, particularly as we have also observed a compaction effect in simulations of the unfolded state of ACBP [36] when using the SHIFT approximation rather than GSE. In villin, the use of relatively short cutoffs or the SHIFT method does not appear to strongly affect the structural properties of the folded state. Likewise, these approximations have little impact on the relative stability of the folded and unfolded states, but this may be a result of the fact that, for villin, the two states have similar sizes (the average radius of gyration of the folded state is ∼9 Å, as compared to 10–12 Å for the unfolded state); effects on relative stability may thus be more system-dependent. More generally, our observations suggest that biomolecular simulations employing cutoffs shorter than 9 Å should be particularly prone to simulation artifacts. We expect that these approximations may be more problematic when subtle details of the distribution of states in flexible systems are of interest. The use of a highly accurate Ewald scheme to account for the long-range electrostatic interactions only partially alleviates the problem, indicating that a substantial contribution to these artifacts comes from the truncation of the Lennard-Jones interactions. It has been suggested that this missing long-range component of the Lennard-Jones interactions can be at least partially accounted for through proper reweighting during data analysis [34], or it could be directly computed during the MD simulation using an Ewald scheme [37]Importantly, the ability to accurately quantify such effects in complex biomolecules allows for more systematic studies of the range of applicability of various approximations, thus paving the way for the development and testing of novel methods that increase computational efficiency, but retain accuracy in the description of relevant biological properties.
